# Pediatricians’ Perspective on the Role of Stepparents in Pediatric Medical Decision-Making

**DOI:** 10.3390/children13020245

**Published:** 2026-02-10

**Authors:** Manon Willekens, Johanna Callens, David De Coninck, Shauni Van Doren, Jaan Toelen

**Affiliations:** 1 Department of Pediatrics, UZ Leuven, 3000 Leuven, Belgium; manon.willekens@student.kuleuven.be; 2 Faculty of Medicine, KU Leuven, 3000 Leuven, Belgium; johanna.callens@student.kuleuven.be; 3 Department of Sociology, KU Leuven, 3000 Leuven, Belgium; david.deconinck@kuleuven.be; 4 Clearinghouse for Self-Help, KU Leuven, 3000 Leuven, Belgium; shauni.vandoren@kuleuven.be; 5 Department of Pediatrics, UZ Leuven and Department of Development and Regeneration, KU Leuven, 3000 Leuven, Belgium

**Keywords:** medical decision-making, medical information sharing, pediatricians, stepparent, children

## Abstract

**Highlights:**

**What are the main findings?**

• Pediatricians consistently distinguish between information-sharing (often allowed) and formal consent (rarely granted) when stepparents accompany a child.
• Decisions about involving stepparents depend strongly on the medical context, relational dynamics, and the perceived vulnerability of all actors involved.

**What are the implications of the main findings?**

• The absence of legal recognition for stepparents leads to inconsistent practices and exposes children, families, and physicians to avoidable risks.
• Clearer guidance could support more consistent, child-centered decision-making.

**Abstract:**

Background/Objectives: Shared decision-making is a central principle in pediatric practice, yet its implementation becomes challenging in the context of alternative family configurations. Stepparents have substantial caregiving roles, but Belgian legislation does not include them in medical information or decision-making authority, creating a gap between legal frameworks and clinical realities. The objective of this study was to explore pediatricians’ perspectives on the involvement of stepparents in medical information sharing and decision-making for minors, and to identify factors influencing whether and how stepparents are included. Methods: A qualitative study was conducted using six semi-structured focus group interviews with 30 pediatricians from six hospitals across Flanders, Belgium. Participants were purposively sampled based on clinical experience. The interviews explored experiences with consent, confidentiality, and stepparent involvement in pediatric care. Data were audio-recorded, transcribed verbatim, and analyzed using constant comparative analysis to identify overarching themes. Results: Three overarching themes emerged. First, the medical context strongly shaped decisions: medical information and minor decision-making were frequently shared, while major decision-making often involved consultation with the legal guardian. Second, relational dynamics, including the quality of the stepparent–child relationship, co-parenting conflict, and physicians’ intuitive assessments, influenced the extent to which stepparents were involved. Third, vulnerability was a recurring theme across all actors: physicians felt legally exposed, children risked fragmented care, legal guardians feared loss of control, and stepparents lacked recognition despite significant caregiving roles. Conclusions: This study shows the importance of a better alignment between clinical practice and legal reality. Aligning legal frameworks with contemporary family patterns may support more consistent, child-centered decision-making in pediatric practice.

## 1. Introduction

Shared decision-making is a central principle in contemporary health care, reflecting a shift away from traditional physician paternalism (i.e., the physician makes medical decisions on behalf of the patient) or complete patient autonomy toward a model that emphasizes a partnership between physician and patient [1,2]. Within pediatrics, however, this model is complicated by the involvement of multiple actors [3,4]. Minors are increasingly recognized as active participants in decisions about their care, yet the legal responsibility for consent typically remains with their parents or legal guardians. This structure assumes a nuclear family (consisting of two biological or adoptive parents and their children), but in practice, modern family arrangements are often far more complex.

The rise of reconstituted families (also called blended or stepfamilies)—which are formed when one or both partners in a couple bring children from previous relationships into a new household, sometimes together with children they have jointly—further complicates this landscape. The 2021 Family Survey of the Flemish Government reported that 13% of families were reconstituted, compared to just over 10% in 2016, marking a steady upward trend [5]. In the U.S., the Survey of Income and Program Participation in 2009 showed that 7.5% of U.S. children younger than age 18 lived with a cohabiting or married stepparent [6]. Steinbach et al. conducted a study across 17 Western countries to examine the prevalence of separated families, finding that single-parent families and stepfamilies constitute between one-third and one-fifth of all families with minor children in most countries [7]. These demographic changes mean that an increasing number of children grow up in households where stepparents are closely involved in their upbringing. Family studies have shown that stepparents often take up a role as active parents and contribute to child-rearing, sharing responsibilities with legal parents [8]. As a result, social parentship is not always solely exercised by biological or adoptive parents [9,10]. While this may already be challenging in the context of child rearing, it adds a significant layer of complexity in the context of medical decision-making.

In Belgium, the legal framework governing medical decision-making of minors is outlined in the Patients’ Rights Act of 2002 [11]. This law grants decision-making authority to those who hold parental authority or legal guardianship. It further stipulates that children should be involved in medical decisions to the extent that their age and maturity allow, with some older minors permitted to act independently if they are deemed competent to assess their own interests [12]. While this legislation provides clarity in most traditional family situations, it does not account for the involvement of other significant caregivers such as stepparents [13]. In the eyes of the law, stepparents remain third parties, regardless of their role in daily caregiving.

Across jurisdictions, the legal authority of stepparents to participate in decision-making for their stepchildren varies considerably. In England and Wales, the Children Act 1989 provides for a *Parental Responsibility Order*, through which stepparents can be granted parental responsibilities via a court order. Australia follows a similar approach: the court can grant stepparents *parental responsibility* through a Parenting Order, which may explicitly include authority over healthcare decisions. This is only granted under specific conditions: the stepparent must demonstrate that the child has lived with them for at least three years, that they are married to or in a civil partnership with one of the child’s biological parents, and that the local authority provides its approval [14].

The absence of formal legal recognition creates difficulties for both families and health professionals. Pediatricians frequently find themselves in situations where stepparents are the ones accompanying a child to consultations, providing medical history, or making practical arrangements for treatment. Yet without legal authority, stepparents cannot give valid consent for procedures or access medical information without the explicit approval of the biological or adoptive parent. This gap between law and clinical reality places physicians in a challenging position, forcing them to balance legal obligations with pragmatic judgments about the child’s best interests.

Limited research has been conducted on the role of stepparents in medical decision- making. Previous work studied the opinions of 2000 Belgian and Dutch parents on the role of stepparents using a case-based questionnaire [15]. The results showed that about half of the participants thought stepparents should receive medical information, though there was less support for allowing them a co-decisive role in medical matters. Respondents did approve of giving stepparents a ‘passive’ role, allowing them to participate in information sharing. However, there was less support for including stepparents in making actual decisions.

The rising prevalence of reconstituted families makes the question of stepparent involvement in pediatric care increasingly important. Pediatricians frequently encounter situations in which a child’s best interests might be served by including a stepparent in decision-making, yet current legislation offers no clear guidance. In the absence of explicit rules, physicians are left to rely on subjective and context-dependent judgments, leading to inconsistencies across individuals and institutions. Such variation can undermine trust: families may feel excluded, while physicians remain uncertain about their professional responsibilities.

Legal and ethical dilemmas in pediatric care most frequently arise in situations involving disclosure of sensitive medical information and long-term or life-altering treatments. Tensions may emerge when family members disagree among themselves or when the accompanying caregiver lacks formal decision-making authority. Previous research has highlighted that uncertainty in such situations underscores the need for clear legal and ethical frameworks, as well as adequate clinical training to support healthcare professionals when navigating complex decision-making contexts.

Given that reconstituted families are not only common but also their number is steadily increasing, it is essential to better understand how pediatricians perceive and address the role of stepparents in medical decision-making. The purpose of this study is to examine pediatricians’ perspectives on stepparent involvement in decisions concerning minors, to identify the factors associated with whether and how stepparents are included, and to provide insights that may inform the development of future guidelines and policies.

## 2. Materials and Methods

*Study design.* A qualitative study design was chosen using focus group interviews. The interview guide (Supplementary Table S1) was developed based on existing literature and the study team’s prior research. It was reviewed and refined by an interdisciplinary expert panel consisting of professors in pediatrics, sociology, and law. In addition, the guide was evaluated by (step)parents, as lay representatives, to ensure clarity, relevance, and comprehensibility from a non-professional perspective. Feedback from both experts and lay reviewers was incorporated into the final version of the interview guide.

The focus groups were moderated by two members of the research team with a background in pediatrics. No indications of inhibited participation or altered group dynamics were observed during these discussions.

The first part of the focus groups explored the socio-demographic characteristics of the participants, the second part explored factors associated with the (non)involvement of stepparents in medical decision-making (consent) and the sharing of our information (confidentiality). Pediatricians across Flanders, Belgium, agreed to participate, eitherphysically or online through Microsoft Teams. The interview lasted for approximately one hour. The interviews were conducted anonymously and voluntarily.

*Participants*. Pediatricians from six different hospitals in Flanders participated, with a broad geographic distribution. A purposive sampling strategy was used to recruit pediatricians with substantial clinical experience in complex family situations. Participants were recruited through professional networks of the research team and participating hospitals. Pediatricians with at least five years of working experience were included, ensuring that they had sufficient hands-on experience with complex family situations. Exclusion criteria were pediatricians with insufficient knowledge of the Dutch language and pediatricians in training. Participating pediatricians were given an informed consent form clarifying the aim, method, and implications of the study; the potential benefits, risks, and drawbacks associated with participation; the possible external funding of the study; and the processing of personal data, as well as the storage and retention period of these data.

*Analysis.* Interviews were audio-recorded and transcribed verbatim with pseudonymization. The recordings were analyzed using the constant comparative analysis method. The interviews were coded in three phases. The first phase of analysis was open coding, where the transcripts were reviewed to identify broader concepts and to achieve data reduction. Secondly, axial coding was performed, where we defined categories and structured them into different overarching themes. We looked at the different characteristics and dimensions of each category and compared the different categories. Thirdly, we used selective coding to form theories out of the concepts and to look for relevant patterns [16].

Two researchers independently coded the transcripts during the open coding phase. Codes were progressively clustered into categories and overarching themes through iterative comparison across transcripts. Coding differences were discussed until consensus was reached. When disagreement persisted, a third researcher was consulted. During data collection and analysis, the research team continuously assessed emerging themes. After six focus groups, no substantially new codes or themes were identified, indicating that thematic saturation had been reached.

*Ethical approval*. The research protocol was assessed by the ethical committee of KU Leuven and was approved (MP024583).

## 3. Results

### 3.1. Participants: Socio-Demographic Characteristics

Thirty pediatricians from six hospitals across Flanders participated in the focus group interviews. Each focus group consisted of four to six pediatricians. Groups included a mix of genders and years of professional experience. The socio-demographic details are visible in Table 1. The median age was 45 years (range 31–65) and the median years of pediatric practice was 20 years (range 6–40), with a female predominance (19 vs. 11). The sample was geographically diverse, with participants from four different Flemish provinces (West-Flanders, Antwerp, Limburg and Flemish-Brabant). This variation ensured that perspectives reflected a broad spectrum of clinical practice settings.

### 3.2. Key Findings

#### 3.2.1. Medical Context

**Figure 1:**
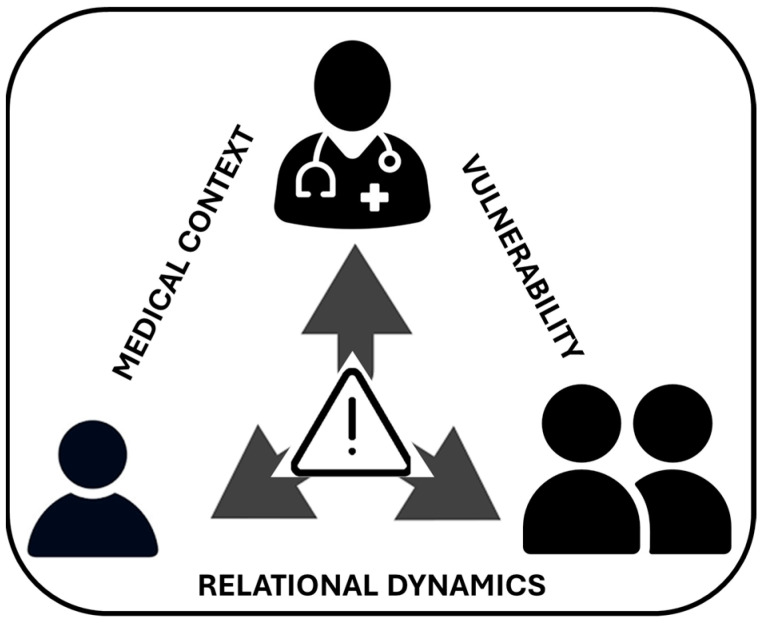
Illustration of the key agents (child, step/parents, physician) and the three main themes (medical context, relational dynamics, vulnerability).

The medical context was an important factor shaping the decision to involve the stepparent. The participants made a clear distinction between providing medical information to stepparents and obtaining formal consent for medical interventions. Figure 1 provides an oversight of the key agents in the paper and the three main themes.

Regarding the sharing of medical information, the participants stated that they almost always informed the stepparent about ‘simple’, everyday medical information (e.g., common cold, and UTIs). They stated it would be very difficult not to give information to the stepparent if they accompanied the child and/or one of the parents to the appointment. However, a major medical problem (e.g., new diagnosis of leukemia or asthma) had to be communicated with the legal parent(s). They were also more cautious in sharing information regarding long-term treatments. Interestingly, the child’s prior medical history was not considered a significant factor in decisions involving stepparents, nor was the age or gender of the child or stepparent. Participants stated that they would only share currently relevant information.

Regarding medical decision-making, several aspects such as invasiveness, urgency, and reversibility of medical intervention were considered pivotal. Participants did not allow stepparents to authorize invasive procedures, including anesthesia or surgical interventions (e.g., appendectomy), while minor interventions, such as a phlebotomy, could be approved by the stepparent. Vaccination, on the other hand, was not something they felt stepparents could decide on, as it is irreversible and also a topic of a broader societal and personal debate. Opinions were more divided regarding the suturing of a skin wound. Some participants argued that consent from a legal guardian was required because it is an invasive procedure. Others noted that it is typically urgent and can be performed as it is in the best interest of the child. Acute minor decisions, such as the start of antibiotics for an infection, were considered acceptable. However, for chronic treatments, for example, prescribing medication for ADHD or cortisone inhalers in asthma, most participants wanted the approval of the legal guardians. Decision-making around imaging was considered more complex due to the radiation risk. As a rule, most participants viewed imaging as a medical decision and therefore felt that a stepparent could provide consent in these cases. An urgent admission into the hospital could be decided by the stepparent if they also discussed it with the legal guardian. In emergencies, physicians emphasized that they acted independently based on clinical necessity, regardless of the presence of the legal guardian or stepparent. Participants frequently referred to acting in what they perceived as the child’s best interests.

Table 2 provides an oversight of the coding terms, categorization of most of the main themes, and citations.

#### 3.2.2. Relational Dynamics

Next to the medical framework, several social and relational factors influenced participants’ decisions. The relationships between the four stakeholders were considered essential.

A long-term relationship between the physician and the family increased the physicians’ willingness to involve the stepparent, as they knew the family situation and could better assess whether it would be appropriate. Both the duration and the quality of the stepparent’s relationship with the child and its parents play a major role: perceived trust and involvement of the stepparent towards the stepchild in the consultation made the participants more willing to involve the stepparent. In contrast, the ongoing conflict between the stepparent and legal guardian reduced the inclusion.

Some participants also considered whether a stepparent was married to or officially cohabiting with a biological parent, though such formal arrangements were not always decisive.

In addition to these relational factors, physicians often relied on their intuition or “gut feeling.” If something about the situation felt “off,” they were more cautious about sharing information with a stepparent. Although such judgments were subjective, they often guided clinical practice.

#### 3.2.3. Vulnerability of Involved Parties

The findings of this study reveal that vulnerability is a recurring theme involving all actors in pediatric decision-making when stepparents are present. The participants often had limited knowledge of the precise legal framework and recognized that no clear legislation exists to guide them. Some even had no knowledge of the specifics of the subject. Many admitted to acting pragmatically and “in good faith,” sharing information with stepparents or allowing them to participate informally to maintain continuity of care, even though they realize that such actions could have legal repercussions. Some participants noted that a strict adherence to the legal framework could result in fragmented or delayed care, thereby harming the child. This, in turn, could also result in legal problems. All participants indicated that they never consult the children for their opinion on the inclusion or exclusion of the stepparent in the decision.

The position of biological parents was at times also considered ‘precarious’. In practice, medical decisions could sometimes be taken by stepparents simply because they were the only ones present at consultations, even though they had no formal authority. This exclusion of the legal parents could undermine parental trust, according to the participants. For this reason, some participants stated that they did not include stepparents in medical decision-making. Others argued that the responsibility lay with the stepparent and the child’s legal guardians. If the guardian did not agree with the stepparent receiving information, he/she should not have sent the stepparent to the consultation. However, some participants counteracted that statement by pointing out that the other legal guardian may not be aware of this situation and that he/she may not agree that the stepparent can make such a decision in the absence of a legal guardian. When participants were asked whether they inquired about who accompanied the child, it became evident that some routinely do so, whereas others only raise this question when concerns or doubts arise. In most instances, they relied on a form of implicit trust, assuming that the accompanying adult had consulted with the legal guardian and was acting with their consent. This assumption was perceived as a means to minimize potential risk for the physician.

Finally, stepparents themselves were perceived as vulnerable figures, as—according to strict implementation of the law—they would be solely reliant on the legal guardian to receive information about their stepchild and not directly from the physician.

#### 3.2.4. Suggestions for Improvement of Practice

When asking the participants how they would adapt the legal framework to better suit the current societal situation and the presence of stepparents in clinical care, opinions were divided. Some participants did not want to change the legal system. They stated that they would rather follow their gut feeling and act in good faith than further complicate it and have to follow legal guidelines. The majority of participants, however, expressed a desire for changes in the legal framework. One frequently mentioned suggestion was the systematic recording of family structures in medical records, including the identification of significant caregivers such as stepparents. This would provide physicians with a clearer context and could provide more consistent decision-making. Another proposal was the creation of a formal status for stepparents or other caregivers, sometimes referred to as a “care confidant” status. This status, with written consent from the legal parents, could grant limited rights to stepparents, such as access to medical information or permission to accompany the child during consultations, making it clearer what could be shared with the stepparent.

## 4. Discussion

This study explored pediatricians’ perspectives on the involvement of stepparents in medical decision-making for minors. Our findings indicate that while physicians recognize the significant caregiving role of stepparents, they are also confronted with uncertainty about the lack of a clear legal framework regarding the involvement of stepparents.

Our results are consistent with earlier work that examined the views of Flemish and Dutch parents on this topic [15]. Overall, participants were in favor of involving stepparents to a certain degree. They made a clear distinction between sharing information and giving consent for medical procedures. Participants considered it acceptable, and often even necessary for the continuity of care, to share medical information with stepparents who were closely involved in the child’s daily care. The type of information, however, played a major role: severe diagnoses were preferably not discussed with stepparents. Medical decision-making—giving consent for a medical intervention or treatment—was based on the severity, urgency, invasiveness, and reversibility of the decision. In previous literature on children’s participation in medical decision-making, a differentiation is often made between minor and major decisions [17,18]. Major decisions could be decisions on interventions like appendectomy or admission in the hospital, whereas minor decisions would include diet changes, start of acute medication such as antibiotics, and ways of delivering medications [19]. Applying such a framework to the involvement of stepparents could help clarify their role without undermining the authority of biological parents.

Besides the medical context, relational dynamics played an important role. The quality of the child–stepparent relationship and the interaction between stepparent and legal guardian shaped participants’ willingness to involve the stepparent. Participants were more inclined to include a stepparent when they knew the family situation and often relied on their intuitive assessment of the social dynamics, referred to as gut feeling. Other studies show that when parents are present at consultation with their child, they often highlight the importance of the parent-physician relationship, where a better relationship increases the involvement of all parties [20]. Research has shown that physicians appreciate the degree to which parents are knowledgeable about and involved in their child’s health problem, which our participants expand towards stepparents as well [21]. Previous social studies about stepfamily relationships by Ganong and Coleman identified six possible types of relationships between stepparent and stepchild, ranging from accepting as a parent to coexisting without further bonds [22]. This classification could be used in further research to decide whether to involve stepparents, based on the role of the stepparent.

A previously unaddressed theme in the existing literature is the vulnerability of the four actors involved: the child, the legal guardian, the stepparent, and the physician. Participants, being the physicians, reported feeling vulnerable due to their limited knowledge of the legal framework and the absence of clear legislation regarding stepparents’ roles. Other studies have already highlighted the fact that physicians often have only limited knowledge of the legal framework regarding medical decision-making [23,24,25] and medical decision-making in minors [26,27]. Acting pragmatically and “in good faith” may serve the child’s immediate needs, but it also exposes physicians to potential legal repercussions. Some participants deliberately chose not to ask who was accompanying the child, reasoning that this would allow them to defend themselves later if problems arose, as they could state that they were unaware the accompanying adult was a stepparent. The vulnerability of the child also emerged as a major concern. Participants feared that strictly adhering to the legal framework could delay or fragment care, particularly when the legal guardian could not be reached. The parents, as legal guardians, were described as vulnerable as well, as decisions could effectively be taken by stepparents simply because they were present. Participants in our study recognized that this ambiguity may threaten parental trust in the medical process. Furthermore, stepparents were portrayed as vulnerable actors because they frequently assumed substantial caregiving responsibilities despite lacking legal recognition. As our results show, their involvement depends largely on the goodwill of physicians and legal guardians.

In the current legal framework in Belgium, there is no official place for stepparents. The law stipulates that a child can only have two legal guardians. Stepparents can only receive parental authority by adopting the child. However, this is not an option when both biological parents want to remain guardians, as one would lose their authority [28]. When participants in our study were asked whether the legal framework should be adapted, their opinions differed. Some expressed concern that implementing a formal legal structure might result in all procedures being handled in a strictly official manner, potentially increasing complexity and even delaying clinical care. Currently, clinicians can pragmatically “look the other way,” acting as if they are unaware of certain legal constraints. While some preferred to retain the existing informal approach, others advocated for establishing a formal “care confidant” status, whereby parents could be granted limited rights through the explicit consent of the legal guardians, recorded in the medical file. Such a system could offer pediatricians greater clarity and facilitate care within a defined legal framework, while ensuring that the child’s best interests remain central.

Further research is required to assess the opinion of other physicians and other stakeholders regarding the involvement of stepparents in pediatric medical decision-making in other countries and continents. Quantitative studies could complement these qualitative findings by identifying patterns across larger populations of physicians. It would also be valuable to compare pediatricians’ views with those of general practitioners, who often interact with families in different contexts and may face different challenges. It could also be interesting to capture the views of children, as previous research has shown that children like to be involved in medical decisions, especially as they grow older [29].

Overall, this study reveals the need for greater alignment between law and clinical reality. At the clinical level, pragmatic strategies were already employed within existing legal constraints, such as relying on implicit trust or prioritizing urgent care. At a systemic level, participants’ suggestions point toward the need for legal and institutional reforms, including clearer documentation of caregiving roles and potential recognition of a “care confidant” status. Distinguishing between these levels is essential, as clinical strategies can be implemented immediately, whereas legal adaptations require broader policy and legislative processes. Without adaptation of current frameworks, physicians will remain caught between legal risk and the obligation to give the best care. Clearer guidelines, potentially supported by legislative reform, are essential to ensure that pediatric practice remains both legally sound and responsive to the needs of rising modern families. The study highlights how the absence of a clear legal framework places all four actors—physicians, children, biological parents, and stepparents—in vulnerable positions, forcing pediatricians to rely on pragmatic strategies rather than transparent guidelines.

While the findings provide valuable insights, several limitations should be acknowledged. First, the study relies on qualitative interviews, which may be shaped by subjective interpretations and the researchers’ own analytical frameworks. Second, the relatively small sample size may have restricted the diversity of viewpoints represented, as participants who agreed to take part may differ systematically from those who declined. Third, the study focuses exclusively on pediatricians, even though general practitioners also frequently engage with stepparents and newly constituted families. Future research could therefore involve a broader range of healthcare professionals and employ quantitative methods to yield more generalizable conclusions. Finally, as with all interview-based research, social desirability bias may have influenced participants’ responses. Pediatricians may have described ethically defensible practices rather than actual behavior in daily clinical practice. This limitation was mitigated by encouraging open discussion and by emphasizing the absence of right or wrong answers, but it cannot be fully excluded.

## 5. Conclusions

Pediatricians experience a complex legal and practical dilemma when dealing with stepparents in medical decisions. The participants stated that they distinguish between information-sharing, which they often considered appropriate, and formal consent, where they made a difference between major and minor decisions. Decisions about involving stepparents were shaped not only by the medical context but also by relational dynamics and vulnerability of the four actors. These findings underscore the necessity for clearer guidelines concerning the roles of stepparents and stepchildren, as well as for the physicians involved. Addressing these uncertainties can enable pediatricians to act in the best interest of the child while ensuring adherence to legal and ethical standards.

## Figures and Tables

**Table 1:** Socio-demographic characteristics (n = 30).

Gender		
Male (N, %)	11	36%
Female (N, %)	19	64%
Age (range)	31–65	
Years of working experience	6–40	

**Table 2:** Framework for qualitative coding.

Vulnerability	Themes	Frequency Indicator *	Quotes
Doctor	Child’s best interest	12	*“In practice, we do share information with stepparents. You assume that the accompanying adult has the best interests of the child at heart.’’*
	Medico-legal risk	7	
	Ignorance of the law	4	*“I can easily say here that I wouldn’t give information to a stepparent, as the law predicts. However, when they are in front of me, trying to take care of their stepchild, I would never send them away without information, unless I have a bad feeling on the situation.”*
	Intention of stepparent	4	
	Unawareness of social situation	2	*“I never explicitly ask who accompanies the child during a consultation. I assume that permission has been given concerning all medical issues.”*
Patient	Central position child	2	*“We also must think about the child. We want to give the best care. If he/she is sick and we would deny care because he/she came with a stepparent; what kind of doctors would we be?”*
	Minor: no autonomy	3	*“As for involving children, a lot depends on the age of the child. Of course, it also depends on the situation, as some children at 14 years old are already much more mature than others.”*
Biological parent	Identification	1	
	Vulnerability	3	
Stepparent	Implicit trust	11	
	Identification of the accompanying person	9	*“If I don’t ask who accompanies the child, and I get in trouble by giving information to the stepparent, I can claim afterwards that I didn’t know who accompanied the child.”*
	Best intentions for the child	7	*“The fact that a stepparent comes along to the consultation shows that they are involved with the child. They are taking care of the child and also just think they are doing the best for that child.”*
	Responsibility stepparent (not doctor)	6	
**Medical** **Context**	Themes	Frequency Indicator *	Quotes
Information	Medical information	5	*“I would make a distinction between information and actually giving consent. I don’t think a stepparent should be allowed to about something like an appendectomy. As soon as you’re doing anything invasive, I believe the biological parent should give permission.”*
Consent	Medical consent	5	
Influencing factors	Urgency (medical need)	13	
	Severity diagnosis	7	
	Chronicity	6	*“It is completely different with chronic medication. I only start Rilatin [long term medication for ADHD] when I have spoken to the biological parents.”*
	Invasiveness	9	
	Complexity	4	*“Of course it also depends a bit on the context. If the stepparent is in front of you and there’s a suspicion of leukemia, you may still ask to call the parents.”*
	Reversibility	1	
	Past medical history	1	
	Relevance of the information	3	
Future	Written consent biological parents	13	*“I think it would be very good if the law is amended and that there is more clarity about what stepparents are allowed to do now. I think this is best done through an explicit written permission from the biological parent.”*
	Care figure in the medical file	6	
	Clearer law	5	*“Current society should adapt to modern family patterns. Almost half of the children we see, are in a composite family. So it’s important that there is some kind of framework for us as a doctor.”*
	No changes of law	3	
**Relational** **Dynamics**	Themes	Frequency Indicator *	Quotes
	Stepparent-child interaction	18	*“I think it is important how the bond between them is: is the stepparent intensely involved? Does he/she know everything about the child? A lot depends on how the interaction between stepparent and child is. This guides you in whether to involve the stepparent or not.”* *“A connection with the child is important. If the stepparent never sees the child or has little involvement, I think it’s weird to give information to that person.”*
	History doctor-family	9	*“In the chronic patients you also know the family situation and you know if the stepparent is involved. In that case, giving information is gray, not black and white.”*
	Social situation	9	
	Duration of presence of stepparent	7	
	Intuition	5	*“It is also important if you have a bad gut feeling in a situation. I think you always follow your intuition a bit.”*
	Sociodemographic (age, gender)	4	

## Data Availability

The raw data supporting the conclusions of this article will be made available by the authors on request.

## Supplementary Materials

The following supporting information can be downloaded at: https://www.mdpi.com/article/10.3390/children13020245/s1, Table S1: framework for qualitative coding.
