# Supplementary material for: Pediatricians’ Perspective on the Role of Stepparents in Pediatric Medical Decision-Making
Source: Children (Basel). 2026 Feb 10;13(2):245. doi: 10.3390/children13020245 (PMC12939647; doi:10.3390/children13020245)
Supplement: Supplementary file 1 [file children-13-00245-s001.zip › children-4099031-supplementary.pdf]

Table S1: framework for qualitative coding

VULNERABILITY		#
**DOCTOR**	Child's best interest	12
	Medico-legal risc	7
	Ignorance of law	4
	Intention of stepparent	4
	Unawareness of social situation	2
**PATIENT**	Central position child	2
	Minor: no autonomy	3
**BIOLOGICAL PARENT**	Identification	1
	Vulnerability	3
**STEPPARENT**	Implicit trust	11
	Identification of accompanying person	9
	Best intentions for the child	7
	Responsibility stepparent (not doctor)	6

MEDICAL CONTEXT		#
**INFORMATION**	Medical information	5
**CONSENT**	Medical consent	5
**INFLUENCING FACTORS**	Urgency (medical need)	13
	Severity diagnosis	7
	Chronicity	6
	Invasiveness	9
	Complexity	4
	Reversibility	1
	Past medical history	1
	Relevance of the information	3
**FUTURE**	Written consent biological parents	13
	Care figure in medical file	6
	Clearer law	5
	No changes of law	3

RELATIONAL DYNAMICS		#
	Interaction stepparent-child	18
	History doctor-family	9
	Social situation	9
	Duration of presence of stepparent	7
	Intuition	5
	Sociodemographic (age, gender)	4
